# Tsunami-Driven Spread of *Toxoplasma gondii* and Other Microbial Pathogens: Implications for Cetacean Health and Conservation

**DOI:** 10.3390/pathogens12040616

**Published:** 2023-04-18

**Authors:** Giovanni Di Guardo

**Affiliations:** General Pathology and Veterinary Pathophysiology, Veterinary Medical Faculty, University of Teramo, 64100 Teramo, Italy; gdiguardo@unite.it

Six years have now gone by since Dr James T. Carlton and coworkers made the scientific community aware of a tsunami-driven spread of marine organisms [1]. The catastrophic earthquake which took place in March 2011 in Eastern Japan was the driver of this unprecedented event. Moreover, such an extraordinary long-distance transfer of mostly invertebrate organisms was largely amplified by micro-nanoplastics, that most likely acted as “rafts” for them [1]. Interestingly enough, the aforementioned phenomenon could also involve microbial pathogens, a number of which are known to impact the health and conservation status of free-living cetaceans [2]. This is the case for *Toxoplasma gondii*, a protozoan and zoonotic agent of major concern [3] infecting striped dolphins (*Stenella coeruleoalba*) and a range of different odontocete and mysticete species. *T. gondii*, an opportunistic pathogen commonly found in *Dolphin Morbillivirus*-infected striped dolphins [4], may also behave as a primary neurotropic agent, thereby causing severe, multifocal, non-suppurative encephalitis lesions, with affected striped dolphins dying in the open sea or otherwise getting stranded alive or lifeless [5]. Despite the marine mammal scientific community’s general consensus on a “land-to-sea transfer” as the most biologically plausible mechanism through which *T. gondii* oocysts may gain access to the sea environment, similarly to other oro-fecally transmitted pathogens [2], this assumption becomes questionable when dealing with striped dolphins and other *T. gondii*-susceptible cetacean hosts living in the open sea [6]. In other words, it is yet unexplained how striped dolphins and other offshore cetaceans may acquire *T. gondii* infection. Different hypotheses have been drawn over time in order to elucidate this intriguing phenomenon, including the occurrence of an “alternate” *T. gondii* marine life cycle [6]. In this respect, provided that “alternate” biological marine cycles have not been hitherto reported to take place, to the best of my knowledge, for *T. gondii* as well as for many other protozoan and non-protozoan pathogens, it would be interesting to investigate whether tsunamis, seaquakes and, more in general, seawater movements may cause long-distance dispersal into marine and oceanic ecosystems of *T. gondii* and other oro-fecally transmitted microorganisms, alongside their vertebrate and invertebrate hosts. Notwithstanding the above, the surface charge of *T. gondii* oocysts, the most environmentally resistant parasite life cycle’s structures, is close to neutral in high-ionic strength solutions mimicking estuarine or marine waters [7], which can enhance oocyst interactions with marine biofilm and algae growing in coastal areas. This process results in oocyst incorporation into marine food webs, with exposure implications to higher tropic level animals and people [7].

Remarkably, *T. gondii* has also been recovered from a number of commercial, edible fish species [8] and, since no clear-cut evidence is hitherto available on fish susceptibility to *T. gondii* infection, these findings of public health relevance provide further support to the assumption that *T. gondii*-contaminated micro-nanoplastics likely served as parasite carriers, after having been eaten by fish. As a matter of fact, an association in the marine environment of the zoonotic protozoa *T. gondii*, *Cryptosporidium parvum* and *Giardia enterica* with polyethylene microbeads and polyester microfibers has recently been described, with more parasitic bodies adhering to microfiber surfaces as compared with microbeads [9]. Within this context, the well-known ability of micro-nanoplastics to serve as “attractors and concentrators” for a wide range of immunotoxic, neurotoxic and endocrine-disrupting, persistent environmental pollutants should be taken into special account, in view of the “top predator” position occupied by odontocete cetaceans along the marine food web, with subsequent bioaccumulation and biomagnification of the aforementioned xenobiotics in their body tissues [10]. Based upon what is reported above, coupled with the opportunistic behaviour of *T. gondii* [4], in a similar fashion to many other protozoan parasites, it is self-understandable how easily odontocete cetaceans carrying heavy amounts of immunotoxic chemical contaminants in their body tissues may become infected by the aforementioned protozoan agent.

Furthermore, the significant role played by the inappropriate disposal of face masks and plastic gloves used in the fight against the pandemic SARS-CoV-2 betacoronavirus over the last three years should be taken into consideration, with this resulting in a growing level of micro-nanoplastics contamination of terrestrial, sea and ocean environments on a global scale. Indeed, should micro-nanoplastics behave as attractors and rafts also for SARS-CoV-2, this could additionally impact the already threatened health and conservation status of dolphins and whales, given the potential susceptibility of several cetacean species to SARS-CoV-2 on the basis of the homology degree of their angiotensin-converting enzyme-2 (ACE-2) viral receptor with the human one [11]. Notably, SARS-CoV-2 can be fecally shed by infected patients for up to 22 consecutive days [12], thus remaining viable and infectious for up to 4.3 and 6 days in sewage and water, respectively [13].

In conclusion, while the herein dealt and supposed “*T. gondii*/micro-nanoplastics synergism” warrants much more focused research in the near future, a scientific evidence-based, multidisciplinary and integrated approach based upon the “One Health, One Ocean” concept/principle would be strongly needed to elucidate the complex dynamics driving the mutual “animal/human host–pathogen–food web interactions” within marine and terrestrial ecosystems.
